# A mixed methods assessment of barriers to maternal, newborn and child health in gogrial west, south Sudan

**DOI:** 10.1186/s12978-016-0269-y

**Published:** 2017-01-19

**Authors:** Lynn Lawry, Covadonga Canteli, Tahina Rabenzanahary, Wartini Pramana

**Affiliations:** 1 Overseas Strategic Consulting, Ltd, 1500 Walnut Street, Suite 1300, Philadelphia, PA 19102 USA; 2 Poeta Esteban Villegas, 12, 12°B 28014, Madrid, Spain; 3 609 Aquaview Drive, Ottawa, ON K4A 4W1 Canada; 4 Canadian Red Cross, National Office, 170 Metcalfe Street, Suite 300, Ottawa, ON CA K2P2P2 Canada

**Keywords:** South Sudan, MNCH, Sexual Gender Based Violence

## Abstract

**Background:**

Health conditions for mothers, newborns, and children in South Sudan are among the worst worldwide. South Sudan has the highest rate of maternal mortality in the world and despite alarming statistics, few women and children in South Sudan have access to needed healthcare, especially in rural areas. The purpose of this study was to understand the barriers to maternal, newborn and child health in Gogrial West, Warrap State, South Sudan, one of the most underdeveloped states.

**Methods:**

A randomized household quantitative study and supplemental qualitative interviews were employed in 8/9 payams in Gogrial West, Warrap, South Sudan. Interviews were conducted with randomly selected female household members (*n* = 860) who were pregnant or had children less than 5 years of age, and men (*n* = 144) with a wife having these characteristics. Non-randomized qualitative interviews (*n* = 72) were used to nuance and add important socio-cultural context to the quantitative data. Analysis involved the estimation of weighted population means and percentages, using 95% confidence intervals and considering *p*-values as significant when less than 0.05, when comparisons by age, age of marriage, wife status and wealth were to be established.

**Results:**

Most women (90.8%) and men (96.6%) did not want contraception. Only 1.2% of women aged 15–49 had met their need for family planning. On average, pregnant women presented for antenatal care (ANC) 2.3 times and by unskilled providers. Less than half of households had a mosquito net; fewer had insecticide treated nets. Recognition of maternal, newborn and child health danger signs overall was low. Only 4.6% of women had skilled birth attendants. One quarter of children had verifiable DPT3 immunization. Five percent of men and 6% of women reported forced intercourse. Overall men and women accept beatings as a norm.

**Conclusion:**

Barriers to care for mothers, infants and children are far more than the lack of antenatal care. Maternal, newborn and child health suffers from lack of skilled providers, resources, distance to clinics. A lack of gender equity and accepted negative social norms impedes healthy behaviors among women and children. The paucity of a peer-reviewed evidence base in the world’s newest country to address the overwhelming needs of the population suggests these data will help to align health priorities to guide programmatic strategy for key stakeholders.

## Plain english summary

Health conditions for mothers, newborns, and children in South Sudan are among the worst worldwide. Few women and children in South Sudan have access to needed healthcare, especially in rural areas. The purpose of this study was to understand the barriers to health for women, newborns and children in Gogrial West, Warrap State, South Sudan, one of the most underdeveloped states.

Female household members who were pregnant or had children less than 5 years of age, and a subset of men with a wife having these characteristics also participated. Overall, 860 women and 144 men participated in the study. More than ninety percent of women and men did not want contraception. There were few if any qualified health personnel to attend to women who were pregnant and therefore pre-and post-pregnancy care was largely unavailable leaving traditional birth attendants as an only option. Women and children were not fully vaccinated, if at all. Both men and women reported sexual violence. Overall men and women accept beatings as a norm.

In conclusion, the health of pregnant women, newborns and children in Warrup State, South Sudan suffers from lack of skilled providers, resources, and distance to clinics. A lack of gender equity and accepted social norms impedes healthy behaviors among women and children. The lack of published data in the world’s newest country to address the overwhelming needs of the population suggests these data will help to align health priorities to guide programmatic strategy for key stakeholders.

## Background

South Sudan, the newest country in the world, continues to have on-going and renewed conflict and is a fragile state. The health conditions for mothers, newborns, and children in South Sudan are among the worst worldwide. South Sudan has the highest rate of maternal mortality in the world, with a maternal mortality rate of 2,054/100,000 live births, and an infant mortality rate of 75/1,000 live births [1, 2]. Despite these alarming statistics, few South Sudanese have access to needed healthcare, especially in rural areas. Less than half of pregnant women can access any type of pregnancy care, especially in rural, dispersed villages [1, 2].

Warrap State has a population of nearly one million [1, 3]. As one of the most underdeveloped states, 91% of the population lives in rural areas [1, 3]. Economic and health indicators for Warrap are below national averages partly due to a health system that has significant limitations in quality and coverage of basic health services [1, 4, 5]. Antenatal care coverage is low (32%) and less than two percent of deliveries occur at a clinic with skilled attendants [4]. Essential newborn care is deemed “non-existent” with only 30% of newborns receiving any type of postnatal care [4, 5]. Infant mortality for Warrap State (139/1000 live births) is twice the infant mortality rate for South Sudan with child mortality (176/1000 live births), 60% higher than national averages and maternal mortality (2173 maternal deaths/100,000 live births), six percent higher than the national averages; thus many infants in Warrap begin their life without their mothers if they themselves survive [2, 3].

There is an absence of evidence-based data, including socio-cultural data to understand all of the barriers to care for maternal, newborn and child health (MNCH) in Gogrial West, Warrap State. In addition, most data are a combination of the entire state and not limited to Gogrial West [1, 3, 5]. To help align health priorities to real needs and to guide MNCH programmatic strategy for key stakeholders in Gogrial West, a household randomized quantitative study and qualitative study were completed to better understand the barriers to care for women, newborns and children.

## Methods

This mixed-methods assessment consisted of a household, population-based quantitative survey and qualitative interviews at the household, community, government and health facility level conducted during a 2-week period in July 2015 in Gogrial West, Warrap State, South Sudan.

### Human subjects protection

Ethics review was obtained from the Ministry of Health (MOH) and conducted in accord with the Declaration of Helsinki [6]. Verbal consent to participate in the study was obtained from all participants (quantitative and qualitative respondents). Every effort was made to ensure protection and confidentiality and to reduce any potential adverse consequence to the participants. Participants did not receive any material compensation. Participants were informed that participation or lack thereof would not affect their access to or the quality of the care they receive, and were explicitly given the right to refuse participation.

### Geographic scope

This multi-stage clustered and randomized household study was conducted to represent all of the population in 8 of 9 accessible payams in Gogrial West, Warrap State, South Sudan. Within the payams, qualitative interviews were conducted to add nuance to the issues found in the household survey.

### Sample size

To determine an appropriate sample size for the quantitative study, we assumed 50% of women in Gogrial West had accessed MNCH services. Therefore, the sample size required to estimate access to MNCH services through a random sample with 95% confidence (margin of error of ±5%) was 770 households assuming a design effect of two.

### Target population

To better define and understand existing barriers to MNCH, household women who were pregnant or who had children under the age of 5 years or a male household member who had a pregnant wife and or children under the age of 5 years were randomly selected to be interviewed for this study.

### Sampling frame

Gogrial West (one of 10 counties in Warrap State) is divided into nine payams and 28 bomas. Population information for the eight of the nine accessible payams was 221,795 persons and 229 “main” villages excluding Akon North, which was inaccessible due to flooding and security [7]. Villages were randomly selected from the MOH approved list of “main village*s*.” Due to the rainy season, many of the village*s* in this list could not be accessed; therefore, only village*s* accessible could be randomized. A list of randomized alternate villages was generated for cases where the randomized village could not be accessed for any reason. For any villages not accessible, the nearest neighbor was used for substitutions. Substitutions for initial randomized villages occurred in 5 of 30 clusters (17%) due to inaccessible roads due to the rainy season.

To allow an approximate probability-proportional-to-size sampling by payam strategy, existing information on the community sizes, as provided by the Republic of South Sudan National Bureau of Statistics, was used to create relative weights to allocate population based proportional distribution of the clusters.

Quantitative participants were selected by via systematic random sampling which utilized a 30 × 30 cluster sampling methodology (30 randomly selected clusters of 30 households) to generalize to the largest population possible. To account for refusals, we targeted our sample size to be 900 households. Households within villages were sampled according to a modified World Health Organization (WHO) EPI Method [8]. Limited time in each cluster (4–6 h) due to security constraints and distance, and poor accessibility and flooding required using purposive and systematic transect selection aimed at covering as many zones as possible. Surveyor transects were selected either from the village center (or a village main accessible point), or from different points of main roads. In villages with large populations, to reach households that were further from village center, surveyors were instructed to skip every second household on their transect.

### Quantitative instrument

The quantitative survey was developed for the Gogrial West context to elucidate the barriers to MNCH and was based on the continuum of care approach that serves as a core principle of programs for maternal, newborn, and child health, and as a means to reduce maternal, neonatal and child deaths [9]. In doing so, these questions would elucidate the barriers to MNCH and highlight where there were barriers throughout the reproductive lifecycle (adolescence, pregnancy, childbirth, the postnatal period, and childhood) and between places of caregiving, which can include households and communities, outpatient and outreach services, and clinical-care settings. The instrument contained 98 questions on respondent demographics, reproductive health, antenatal care service use, postnatal care, breastfeeding, child health, sexual gender based violence (SGBV), and opinions. Given the surveyor’s preference for English (and the inability of the surveyors to write in Dinka), only key words were translated into Dinka. The instrument was verbally translated into Dinka by the group and individuals were tested to ensure standardization. They were monitored by two Dinka speaking individuals. Observed role-play and field testing was designed to both ensure functional equivalence of the questions across the two languages as well as adjust for cultural issues.

### Qualitative instruments and sampling

Qualitative interviews added nuance to the issues identified in the quantitative instrument and were administered by researchers with the aid of a translator. The qualitative instrument contained 12 open-ended questions relating to barriers to care with regard to MNCH, causes of malnutrition in communities, roles within families, decision-making, cultural norms, traditional practices and community needs. Interviews were used to identify common themes regarding gender barriers and cultural norms affecting MNCH within the context of Gogrial West, Warrap State. Convenience sampling was used to allow flexibility in accessing individuals who might be important for informing the quantitative data. As these individuals were sampled and used to add nuance and context to the quantitative survey, there was no set number of individuals needed. A total of 72 key informants and 25 community members-10 males and 15 females, were interviewed. Interviews included women and men at the village level, key informants including community leaders/healers (religious/tribal/ethnic), beneficiaries, NGO personnel, Ministry of Health, United Nations, World Bank, healthcare providers, traditional birth attendants (TBAs), and community health workers (CHWs).

The information from these individuals was handwritten in the field and used to provide additional insights into the themes and findings that emerged from the quantitative survey. Handwritten notes were then transcribed into a PDF form on the computer that could easily summarize narrative testimonies into themes within all the interviews transcribed.

### Training

Four women and six men to serve as surveyors were recruited from the Warrap State. Training included a 4-day training with a standardized curriculum including observed role-play and field testing [10]. Supervisors were present continuously throughout the survey period to ensure data quality and veracity.

### Household sampling

Teams arrived early in the day and in some cases interviewed respondents while they worked in gardens. Upon arrival to a village, the boma chief was contacted to request permission for the survey. A small token of appreciation was extended to the chief and in return they asked residents to be available for the survey. Surveyors conducted a one-on-one, anonymous interview with a randomly selected male or female household member in a setting that afforded privacy and confidentiality. If no one met the criteria, the next household to the right was surveyed. If only one sex or adult was present at the time of the household visit, that person was interviewed regardless of sex. Surveyors were not assigned by sex. Interviews took approximately 30–40 min and were conducted in Dinka. Surveyors completed 6–7 surveys per day. All surveys were reviewed for completeness and correctness at the end of each cluster.

### Statistical analysis

Quantitative household data were analyzed using XLSTAT. Analysis involved the estimation of weighted population means and percentages (descriptive statistics and cross-tabulations), using 95% confidence intervals and considering *p*-values as significant when less than 0.05, when comparisons by age, age of marriage, wife status and wealth were to be established. Weighting was necessary to keep the per payam proportion of male (of ages 20 years or above) and female (of ages 15–49 years) population in our collected sample equal to that of the actual population in Gogrial West (excluding Akon North). Wealth was measured in a comprehensive and context adapted manner, through the use of both a wealth indicator and wealth groups constructed through a Principal Components Analysis and a k-means classification respectively. The latter were calculated based on size of crops, livestock, mobility and communications assets and monthly income of households.

Qualitative narratives transcribed onto a standardized PDF form were compiled into a master Excel spreadsheet. Themes were hand coded by issues identified in the household quantitative study, analyzed and used to add nuance to the quantitative data.

### Role of the funding source

The funder of the study had no role in study design, data collection, data analysis, data interpretation, or writing of the report. The corresponding author had full access to all the data in the study and had final responsibility for the decision to submit for publication.

## Results

### Demographics

**Fig. 1:**
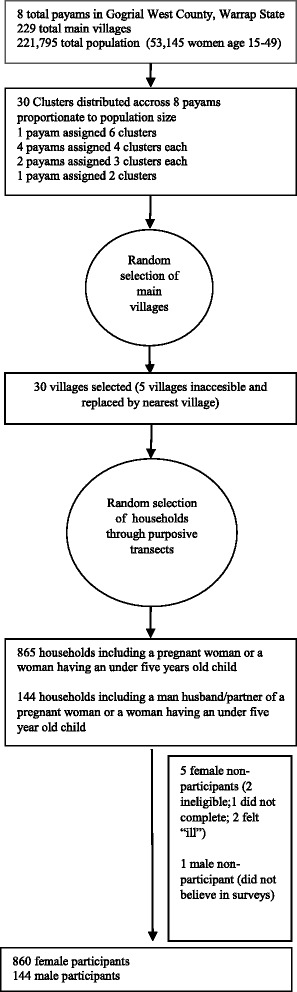
Flow of Participants

A total of 860 households consented to the study (860 women and 144 men) with a response rate of 99.4% among women and 99.3% among men (Fig. 1). The average age of female respondents was 28.5 years and 40 years among males (Table 1). Women had less than 1 year of formal education (0.3 years), whereas men had just over a year of formal education (1.1 years). Qualitative interviews revealed that once married, women and/or girls do not continue in school. Agreements are made in some circumstances to allow continued education after marriage but in practice most husbands do not pay school fees. In addition, although women and/or girls may be in school after marriage, as soon as she is pregnant, she can no longer attend school.

**Table 1:** Weighted Demographic Characteristics for 1004 Respondents

Characteristic	N/Total Respondents, Female	Females Weighted % (95% CI)	N/Total Respondents, Male	Males Weighted % (95% CI)
Age^a^, mean, y	852/860	26.0 (16.0,47.0)	142/144	40.0 (18.0,75.0)
Education				
None	833/860	93.0 (91.2,94.7)	137/144	77.7 (70.8,84.7)
Years (formal)	833/860	0.3 (0.2,0.4)	137/144	1.1 (0.7,1.6)
Unable to read	803/860	95.4 (94.0,96.9)	136/144	83.3 (77.0, 89.6)
Marital status	851/860		144/144	
Never				0.8 (0.0,2.2)
Married		95.5 (94.1,96.9)		97.6 (95.0,100.0)
Widowed		3.8 (2.5,5.1)		0.8 (0.0,2.2)
Other^b^		0.7 (0.1,1.2)		0.9 (0.0,2.4)
Wife number	850/860			
First		62.4 (59.2,65.7)		
Second		21.1 (18.4,23.9)		
Third or greater		16.5 (14.0,19.0)		
Number of wives			137/144	
Mean				1.8 (1.7,2.0)
None				0.8 (0.0,2.3)
One				43.4 (35.1,51.7)
Two				35.1 (27.1,43.0)
Three or more				20.7 (14.1,27.7)
Bride Price^c^	819/860	25.9 (25.0,26.9)		
Age of marriage, y	819/860	17.7 (17.6,17.9)		
Occupation	843/860		143/144	
Farmer		75.5 (72.6,78.4)		63.2 (55.3,71.1)
Homemaker		15.7 (13.2,18.2)		4.6 (1.2,8.1)
Small business		5.9 (4.3,7.5)		13.4 (7.8,19.0)
Government		0.2 (0.0,0.5)		10.9 (5.8,16.1)
Other^d^		2.7 (1.6,3.8)		
Monthly income, mean USD	763/860	11 (10,12)		
Water				
Enough available^e^	840/860	68.4 (65.2,71.5)		
Distance to source (mins)	787/860	32.7 (30.0,35.3)		
Time fetching/d (hrs)	765/860	2.6 (2.5,2.8)		
Fetched water while pregnant	844/860	97.4 (96.3,98.4)		
Water source while pregnant^f^	857/860			
Protected source		83.9 (81.5,86.4)		
Unprotected source		16.1 (13.6, 18.5)		

^a^Qualitative interviews revealed that female respondents did not know their age. Respondents stated “unless a girl or woman is in school, she may not know her age” or “you will need to ask my mother;” therefore, women’s age is likely an estimate

^b^Other: living with partner, spouse missing/killed in conflict/divorced/separated

c This estimate assumes one cow has a value of 250 USD, and was equivalent to 5 goats or 12 sacks of durah which was counted as half a cow. This price represent the price paid in cows to the wife’s family for marriage

^d^Other: day labor, student

^e^Enough, yes or no for drinking, washing hands, bathing for everyone in the household

^f^Protected water source: borehole, protected well/spring; Unprotected water source: Unprotected well, Unprotected spring, River/stream, Lake/pond, Rain catchment

More than 97% of men and 95% women were married. First wives predominated the sample and men reported an average of two wives. The average bride price was 26 cows (equivalent to 6,500 USD), and all women married before 18 years. More than 75% of women and 63% of men stated their occupation to be farming with an average monthly household income of 11USD. Women stated the average time to a water source for collection was 33 min and they spent more than two and a half hours each day fetching water. Most women (83%) reported a protected water source while they were pregnant. At the time of the survey, 18.7% of women were pregnant (Table 2).

**Table 2:** Weighted Characteristics for MNCH Continuum of Care

Characteristic	N/Total Respondents, Female	Females Weighted % (95% CI)	N/Total Respondents, Male	Males Weighted % (95% CI)
Pregnant/Wife pregnant	845/860	18.7 (16.1,21.3)	144/144	25.7 (18.5,32.8)
Contraception				
Met Need	807/860	1.2 (0.4,1.9)	143/144	1.2 (0.0,3.1)
Using any method	817/860	2.2 (1.2,3.2)	136/144	2.5 (0,5.1)
Do not want	844/860	90.8 (88.9,92.8)	143/144	96.6 (93.6,99.6)
Barriers to use	838/860		142/144	
Not traditional		39.9 (36.6,43.2)		49.6 (41.4,57.8)
Spouse		20.8 (18.0,23.5)		8.6 (4.0,13.2)
Nothing		16.9 (14.3,19.4)		31.1 (23.5,38.7)
Availability		12.6 (10.3,14.8)		11.6 (6.3,16.8)
Cost		3.3 (2.1,4.5)		0.8 (0.0,2.3)
Mother-in-law		0.3 (0.0,0.6)		
Don’t know		1.6 (0.7,2.4)		0.7 (0.0,2.0)
Other		4.7 (3.3,6.2)		
Care During Pregnancy	850/860			
Antenatal care, mean^a^		2.3 (2.2,2.4)		
At least one visit		12.2 (10.0,14.4)		
4 visits		14.2 (12.0, 16.7)		
By skilled provider		14.2 (11.8,16.5)		
Tetanus (TT2+)^b^	340/360	31.4 (26.5,36.4)		
Mosquito nets				
1 or more nets in household	858/860	47.4 (44.1,50.8)		
Insecticide treated net	836/860	21.5 (18.7,24.3)		
Household member slept	836/860	39.4 (36.1,42.7)		
Under net night before survey				
Treated net use when pregnant	155/860	9.6 (4.9,14.2)		
Recognition of ≥ 3 pregnancy danger signs	837/860	49.6 (46.2,53.0)	139/144	53.4 (45.1,61.7)
Postnatal Care				
Within 2 days of birth^c^	821/860	7.9 (6.1,9.8)		
By skilled provider		8.0 (6.1,9.8)		
Danger sign recognition (>3)				
Upper respiratory infection	857/860	32.0 (28.9,35.1)	144/144	19.3 (12.8,25.7)
Malaria	854/860	60.1 (56.8,63.4)	144/144	67.2 (59.5,74.8)
Diarrhea	854/860	48.5 (45.2,51.9)	144/144	56.5 (48.4,64.6)
Malnutrition	851/860	38.5 (35.3,41.8)	144/144	39.5 (31.5,47.4)
Newborn	851/860	45.8 (42.5,49.2)	144/144	42.9 (34.8,51.0)
Essentials of newborn care	855/860	49.2 (45.9,52.6)	143/144	44.2 (36.1,52.3)

^a^Average number of visits for care while pregnant

^b^Two doses of tetanus toxoid vaccination verified by an immunization card

^c^Postnatal care for mother and or baby 2 days after birth

### Contraception

More than 90% of women and men (96.6%) did not want contraception. Therefore, of the remaining 10%, only 1.2% of women aged 15–49, who wanted family planning, were able to meet their need for family planning (Table 2). The most common barriers included, lack of desire for contraception, contraception is “not traditional”, spouse’s refusal to allow use, and the lack of availability. Women married before 18 years, were less interested, or desired using contraceptives compared with women who married after 18 years [6.4%; 95% CI (3.9,8.9%) vs 9.3%; 95% CI (6.6,12.0%), *p* < 0.05]. A 21-year-old woman stated: “…men value themselves by the number of cows and wives they have; women by the number of children.” For men, the reasons were not about worth but for fame “…Our name will be known and we [our name] will not disappear…if we marry a girl who is young, we are assured to have many children and we will be famous.”

### Care during pregnancy

On average, pregnant women presented for antenatal care (ANC) 2.3 times and rarely by skilled providers (Table 2). Only 31% of women had verifiable (card present) two doses of tetanus toxoid immunization (TT2+). Forty-seven percent households had a mosquito net and even fewer (21.5%) had an insecticide treated nets (ITN). Less than 10% of pregnant women slept under ITN mosquito nets. For household members, only 39.4% slept under a mosquito net if it was present in a household. Pregnancy danger sign recognition (≥3 signs) was 53.4% among men and 49.6% among women. Qualitative interviews revealed that ANC were primarily sick visits. Women stated costs of services ranged from 2SSP to 150 SSP (20 cents to 15 USD).

### Childbirth and postnatal care

Just over 75% of women had a birth attendant, however only 4.6% had a skilled attendant at delivery. Most women (51.6%) used a TBA/village midwife (Table 2). Wealth had no significant effect on access or use of MNCH services. Interviews revealed clinics were not well-equipped for births, as they did not have skilled attendants for delivery. In addition, interviews indicated cultural preferences for home deliveries. TBAs were both trained and untrained; many used unsterile techniques. Village surgeons (*Arets*) were consulted for obstructed births. *Arets* used a scalp hook to dislodge live infants, an unsterilized spear for dismemberment and removal of stillbirths. Spiritual and traditional leaders treated a variety of maternal health issues around pregnancy and childbirth, most often through casting spells and medicinal roots. Immediate breastfeeding rates were high (98.2%) and exclusive rates low (5.1%). Interviews revealed that only women who were ill or had “no milk” did not breastfeed. A variety of supplements were used for infants less than 6 months; water being the most common. Commonly held myths regarding nutrition prohibit breastfeeding women from consuming groundnuts. Children are not fed eggs or chicken as the belief is that children will not talk or will have elevated blood pressure if chicken is consumed. In general, men determine breastfeeding length. Women believe that malnutrition results from engaging in sex while breastfeeding, therefore, many try to abstain from sex while breastfeeding but struggle as decision-making power lies with the male partners. Only 7.9% of women accessed postnatal care, which was largely done by unskilled providers. The danger signs of childhood illnesses and essential newborn care were largely unknown among male and female respondents.

### Child health

Twenty-four percent of children had verifiable DPT3 immunization. Most children with suspected pneumonia were treated however interviews revealed “treated” was at times by traditional/spiritual healers. The main barriers for healthcare were cost, distance and lack of transportation. Both women and men shared responsibility for access to immunizations. Beatings as punishment, were used routinely by parents and older siblings (Table 3).

**Table 3:** Weighted Characteristics for Child Health

Characteristic	N/Total Respondents, Female	Females Weighted % (95% CI)	N/Total Respondents, Male	Males Weighted % (95% CI)
Children <2 years, DPT3	847/860			
None		63.9 (60.7,67.1)		
At least one child		24.5 (21.6,27.4)		
No children <2 years		11.6 (9.4,13.7)		
Girls	55/860	49.0 (35.9,62.1)		
Boys	216/860	64.9 (58.6,71.3)		
Treated for suspected				
pneumonia (<5 years)	848/860	67.9 (64.8,71.1)	144/144	67.4 (59.7,75.1)
Barriers, access to care	840/860			
Cost		43.6 (40.2,46.9)		
Distance		28.0 (25.0,31.0)		
Lack of transportation		12.7 (10.5,15.0)		
Permission, husband		7.5 (5.7,9.3)		
Too busy/kids at home		3.0 (1.8,4.1)		
Clinic cannot help		2.4 (1.4,3.4)		
Newborns do not need care		1.3 (0.5,2.1)		
Other^a^		1.2(0.4,1.9)		
Husband/I taken children to clinic for immunizations	852/860	65.8 (62.6,68.9)	143/144	74.6 (67.5,81.7)
Violence				
Use beatings for discipline	848/860	85.5 (83.1,87.9)	142/144	87.3 (81.9,92.8)

^a^Other: permission from mother-in-law, concerns about security and don’t know/remember

### Violence

Twenty percent of women and 16% of men reported beatings and insulting, degrading behavior from family on a regular basis (Table 4). Seven percent of women reported pregnancy loss due to violence, which was largely assault (16.9%), and domestic violence (20.9%). Men (5.1%) and women (6.4%) reported forced intercourse. Overall there is a cultural acceptance that a woman’s value is determined by the number of children she bears in addition to men’s views that sex is an entitlement and women can be punished for refusing sex. Women married at young ages were less likely to agree to forced sex but believed they did not have a right to refuse sex and more likely to believe beatings were acceptable. Interviews revealed other forms of community violence such as abduction and rape of girls refusing marriage known as an “elopement”, and beliefs by TBAs that prolonged labor (8 h) is a result of infidelity, and the name of the person must be given to the husband to save the life of the mother and baby. Women were not protected by older women, even if a young girl did not want marriage or sex, mothers would bring the child, against her will, to the husband. A village elder stated, “…if a girl refuses to have sex when she is married and the marriage is not official, we go to the girl’s family to tell her mother and her aunties. If she runs away and is at home, her mother and aunties will bring her back and stand outside the house to make sure the girl does what she is supposed to do…we even know aunties who have held the girl down in our village…”

**Table 4:** Weighted Characteristics for Violence and Gender Opinions

Characteristic	N/Total Respondents, Female	Females Weighted % (95% CI)	N/Total Respondents Male	Males Weighted % (95% CI)
Beatings by spouse	850/860	20.2 (17.5,22.9)		
Degrading, insulting behavior from family on a regular basis	830/860	20.3 (17.6,23.1)	139/144	16.7 (10.5,22.9)
Age of marriage > 18 years	429/860	26.9 (22.7,31.2)		
Age of marriage < 18 years	386/860	13.5 (10.0,16.9)		
Forced to engage in sexual intercourse against will	845/860	6.4 (4.7,8.0)	143/144	5.1 (1.5,8.7)
Age of marriage > 18 years	429/860	8.9 (6.2,11.6)		
Age of marriage < 18 years	386/860	3.9 (2.0,5.9)		
Pregnancies lost due to violence	820/860	7.4 (5.6,9.2)		
Type of violence	55/860	16.9 (7.0,26.8)		
Direct Assault		1.9 (0.0,5.4)		
Mob violence		6.4 (0.0,12.8)		
Conflict		20.9 (10.2,31.7)		
Domestic violence		18.9 (8.6,29.3)		
Neighbor violence		8.9 (1.3,16.4)		
Prefer not to say		26.1 (14.5,37.7)		
Don’t know/remember				
Husband has a right to beat a wife if she disobeys	849/860	68.6 (65.5,71.7)	144/144	61.4 (53.5,69.4)
Age of marriage > 18 years	429/860	64.2 (59.7,68.8)		
Age of marriage < 18 years	386/860	75.2 (70.9,79.5)		
I/my wife has a right to refuse sex	853/860	58.5 (55.1,61.8)	142/144	52 (43.8,60.2)
Age of marriage > 18 years	429/860	48.2 (43.5,52.9)		
Age of marriage < 18 years	386/860	35.0 (30.3,39.8)		
My husband has/I have the right to have sex with me/my wife even if I don’t/she does not want to	855/860	36 (32.8,39.2)	142/144	36.6 (28.7,44.5)
Age of marriage > 18 years	429/860	42.1 (37.4,46.8)		
Age of marriage < 18 years	386/860	31.2 (26.6,35.8)		
My husband/I would allow me/my wife to use birth control if I wanted to use it	840/860	9.8 (7.8,11.8)	144/144	9.5 (4.7,14.3)
My husband/I would use condoms if I/my wife asked	846/860	7.8 (6.0,9.6)	144/144	6.0 (2.1,9.9)

## Discussion

The continuum of care approach for MNCH includes integrated service delivery for mothers and children from pre-pregnancy to delivery, the immediate postnatal period, and childhood to reduce maternal, neonatal and child deaths and improve health [9]. Gogrial West had significant institutional and social barriers to MNCH based on this approach.

### Brief summary of findings

Barriers to MNCH were more than lack of access to focused ANC. Women in Gogrial West had minimal education and little desire for contraception which impact maternal health and mortality [11, 12]. ANC was largely non-existent and consisted of sick visits instead of focused ANC and clinical facilities with a dearth of appropriate skilled healthcare workers to administer ANC. Mosquito net use, although high for the area, still represented only a small proportion of households putting women and children at risk, and especially pregnant women at risk for malaria. Immunization status of both women and children was low. Exclusive breastfeeding rates of 5% put a significant number of newborns and infants at risk for diarrhea and death [13, 14]. The overall lack of knowledge of danger signs, low use of skilled attendance at birth and the preferred use of largely untrained TBAs put women and newborns at significant risk in Gogrial West and represented barriers to MNCH that given the current situation will be difficult to change [13, 14]. In addition, a dearth of facilities that meet basic or emergency obstetric (EmOC) care criteria, lack of transportation and access, especially during rainy season, for obstetric emergencies leave women at the hands of untrained birth attendants and delivery at home. The time and distance needed to fetch clean water, especially while pregnant and the overall lack of clean water will continue to hamper MNCH. Finally, the presence of systematic and accepted violence in the community, largely addressed to women and children due to negative social norms will continue to add to barriers to health for women, newborns and children.

### Context of findings

#### Contraception

Contraception as an effective primary prevention strategy decreases maternal mortality 44% and satisfying the unmet need for contraception another 29% [12]. Nationally, 8% of overall women use contraception. Less than 1% (0.3%) of women in Warrap State use any method of contraception and less than 3% of men and women in our study used contraception [1, 3]. Barriers to the use of contraception included long held social norms,women’s inability to discuss these issues, and men’s control of contraception among their wives. The difficulty for women to access contraception will hamper improvements in MNCH [12].

#### Danger sign recognition

Nationally, only 4% of women could recognize newborn and obstetric danger signs [1] compared with our study population where 45.8% of women and 42.9% of men were able to recognize newborn danger signs and 49.6% of women and 53.4% of men were able to recognize obstetric danger signs. Given the use of community health workers in previous programs and the need for communities to care for themselves because of the lack of clinical facilities, it is likely the recognition of danger signs was, in part, due to education from CHWs. In addition, the strong need for parents to care for their children in a setting where health care facilities could not meet their needs may have played a part in awareness despite available care.

#### Antenatal care

Focused ANC is essential to decrease newborn mortality [13, 14]. ANC (at least four visits) in Gogrial West was six percentage points lower than the national average (20.5 vs. 26%) [1]. Skilled birth assistance was found to be more than 5 percentage points lower than the national average (4.6% vs. 10%) and 3 percentage points higher than Warrap State [1, 3], whereas globally, at least 61% of women receive at least four ANC visits [14]. Within Sub-Saharan Africa, 50% of women have skilled attendants at delivery, 4.6% in this study, 8.5% for Warrap and 19% nationally [1, 3, 14]. Although non-use of ANC has been linked to the poor recognition of pregnancy danger signs in South Sudan [15], our study found close to half of women and men knew >3 pregnancy danger signs, which suggests in Gogrial West knowledge of these danger signs was not related to care-seeking. In Gogrial West, access and or use of ANC is not feasible given fees charged at the clinic level and the distance to clinics. The overall lack of roads, limited access to clinical facilities for a large portion of the year, no ANC at the clinic level, no skilled providers and no EmOC facilities will continue to hamper the use of ANC for women in Gogrial West and continue to put MNCH at risk.

#### Postnatal care

Eight percent of newborns received postnatal care at any time, which is 5 percentage points higher, but still severely lacking than the average for Warrap State [3]. Postnatal care data is not available for South Sudan; however, from qualitative interviews it was clear barriers to post-natal care was an overwhelming misunderstanding of its importance to health, inability to leave the care of children to others to seek care, distance and time to clinical services, especially during the rainy season.

#### Breastfeeding

The prevalence of early breastfeeding (within one hour) in this study was 98.2% percent, which is nearly double the African prevalence of 50% [14]. Data, other than our study, from South Sudan on early initiation of breastfeeding is unavailable. It is estimated that 16% of neonatal deaths could be averted if all infants were breastfed exclusively from the day of their birth and 22% averted if breastfeeding started within the first hour [13, 15, 16]. In Gogrial West, only 5.1% of infants are exclusively breastfed compared to 20% of infants in Warrap State and 28% of infants nationally [1, 3]. The common belief that water and cow’s milk can be administered to babies under 6 months in addition to the belief that sex cannot happen if a woman is breastfeeding will continue to shorten the exclusive breastfeeding period and put newborns and infants at risk.

#### Immunization

Only a quarter of children had verifiable immunization for DPT3 in Gogrial West and were fully immunized. This was however, higher than the national average of 13% and 21 percentage points and higher than state averages of 3.2% [1, 3]. Previous vaccination campaigns and CHWs may have helped to ensure children in Gogrial West were vaccinated. Three-quarters of children not having full vaccination will continue to create a barrier to healthy mothers and children and will require vaccination campaigns to decrease the risk of neonatal, infant and child deaths from preventable diseases. Given the unhygienic practices used by TBAs and the risk for tetanus for mothers and neonates, this will be especially important.

#### Violence

Finally, when looking at barriers to MNCH, sexual and gender based violence (SGBV) plays a significant role in health of mothers and newborns including increasing the risks of HIV, sexually transmitted diseases, unintended pregnancies, unsafe abortions, and the health of mothers and newborns [17–19]. Global prevalence figures suggest that 35% of women worldwide have experienced domestic or SGBV in their lifetime [17]; 20% reported among reproductive age women in South Sudan [1]. In our sample, 20% of all women reported several forms of SGBV including forced intercourse and domestic violence. However, many women believe that their role is to provide sex even if they do not want it suggesting that SGBV including rape is normalized. SGBV has a profound effect on MNCH and pregnant women are at the greatest risk and associated immediate risks to the health of mothers and an unborn child [18, 19]. Children of abused women have a higher risk of death before reaching age five and violence during pregnancy is associated with low birth weight of babies [19]. Women who reported pregnancy losses due to assault and domestic violence, and acceptance of beatings by husbands for failure to “do things right” (68.6% by women in Gogrial West versus 79% nationally) suggests normalized violence [1, 3]. The close to half of women married before age 18, constitutionally considered SGBV, with rates as high 47% in Gogrial West and within 45% in South Sudan, despite a ban in early marriage in the constitution [20, 21], will continue to be at risk and barrier for MNCH especially with regard to obstructed births [22]. Furthermore, the association in our data between early marriage and danger sign recognition, whereby women married after 18 years were more likely to be aware of health danger signs than early married girls, will play a continued negative role for healthy women and girls.

### Programmatic implications

#### Care for women and girls before pregnancy

With higher education, women tend to have fewer children and space births more widely, which reduces maternal and child mortality [11, 12, 23]. Education is lacking in Gogrial West and requires a concerted effort to ensure women and children are educated. Early grade reading programs in bomas and subsidized school fees will be necessary to address some of the barriers to education, but negative social norms regarding education for women and girls must be addressed in order to make any meaningful changes for the future. Improved literacy and education levels will also have a positive effect on family planning [11, 12]. Within the context of Gogrial West, effective contraception as a cost effective intervention to save lives and improve child health may be difficult to implement. The overall lack of education and literacy among women and girls, desire to use contraceptives, long held social norms whereby children are needed for defense of clans, work, as well as infamy for males, women’s inability to discuss these issues with husbands, and men’s control of contraception among their wives will impede programs to supply contraceptives. Barriers to both education and contraceptive use through negative social norms will require collaboration of traditional leaders and time to enact positive social norm behavior change.

#### Care during pregnancy

ANC was largely non-existent and consisted of sick visits instead of focused ANC. Other barriers to ANC included a belief that clinics did not have adequate staff or supplies. The cost of services and the distance to clinics (especially during the rainy season) for many was an impediment. Despite the MOH policy of free healthcare providers and facilities were charging “under the table” for services making ANC impossible for most. As a part of focused ANC, intermittent treatment for malaria in pregnancy (IPTp) and the distribution of ITN can reduce newborn deaths 37–71% [9]. The procurement and use of bed nets was limited and needs to be increased. Interventions such as ITN distribution is more effective and cheaper than case management of malaria during pregnancy. However, understanding the danger signs of malaria and the skills to refer and treat women with uncomplicated and/or complicated malaria is also necessary whether the provider is a community-based provider or at the health facility level. Although iron and folic acid supplementation, tetanus toxoid immunization, syphilis testing and treatment and counseling of maternal and infant nutrition, and IPTp are best managed at a facility level, they can also be managed, with the exception of syphilis testing, at the community level with trained community health personnel [14]. Given the low rates of TT2+ and untrained traditional birth attendants (TBAs) using sorghum stalks, TBA’s and *Aret’s* using unsterilized knives and spear heads and the lack of clean birth kits and/or Chlorhexidine, an intervention to cover this gap is likely to decrease a significant number of deaths among mothers and newborns [9, 13, 14].

#### Child birth and postnatal care

The Ministry of Gender and Child Social Welfare (MOGCSW) recognizes that there is no alternative to TBAs and recommends that TBAs be educated and equipped, in the short term to aid in safe deliveries until a cadre of skilled healthcare providers can be available [24, 25]. Infant mortality can be improved by 55–87% with breastfeeding, and 27% with community based pneumonia case management [9, 13, 14]. Although immediate breastfeeding rates are high, rates of exclusive breastfeeding, the lack of danger sign recognition and subsequent treatment, immunizations and knowing the essentials of newborn care suggests that education and a behavior change communication approach and community case management are imperative in Gogrial West. An improvement in the numbers of skilled birth attendants and well-supplied facilities will be difficult to acheive in the near term and will require significant funding and country buy-in.

#### Cross-cutting programs

Without access to water, sanitation and hygiene (WASH), women and girls are exposed to infections, suffer a lack of dignity, and have a higher risk of child and maternal mortality due to diarrheal diseases in addition to injuries from carrying water [9, 26, 27]. WASH programs for Gogrial West need to address gender roles for fetching water, especially when pregnant, safe drinking water, and education regarding danger signs for diarrhea. With regard to SGBV, the Ministry of Gender and Child Social Welfare (MOGCSW) recommends a national policy to address SGBV; however, funding, support and a will to address these issues may hinder such a goal [24, 25]. Addressing early marriage and the inability to negotiate sex, education, prevention and testing are important to decrease the 75% greater risk of HIV among girls who marry early [28]. Children are, on paper, protected from early marriage however, enforcement of the laws are rare [20, 21]. Finally social norms and values influence how women and children are protected or harmed [29]. With women’s acceptance of IPV as a norm, barriers to MNCH will continue until negative social norms are addressed [29].

## Conclusion

Barriers to MNCH in Gogrial West are far more than the lack of antenatal care and are multifactorial in nature. Funding constraints, an overall lack of skilled providers, lack of resources, distances of communities to clinics, rainy season flooding, poor roads and no vehicles will affect adequate MNCH in this remote area. Furthermore, a lack of gender equity and accepted negative social norms impedes healthy behaviors among women and children and presents a difficult barrier to overcome. The low availability and use of skilled attendance at birth and the preference for untrained TBAs will put women and newborns at significant risk in Gogrial West. The lack of knowledge of danger signs and an understanding of when to seek care, lack of exclusive breastfeeding and a dearth of facilities that meet basic or emergency obstetric (EmOC) care criteria and lack of transportation and access, especially during rainy season, leave women at the hands of untrained birth attendants and delivery at home. Finally, the presence of systematic and accepted violence in the community, largely addressed to women and children and due to negative social norms, will continue to add to barriers to health for women, newborns and children. Changes to these norms, will require engagement and consensus over time to make positive changes in social norms in order to make strides in the improvement of MNCH.

### Limitations

Flooding during the rainy season limited access to areas randomly chosen for surveying. Akon North was not represented in this study as access required movement into volatile areas and the time to reach Akon North did not support ICRC guidelines for security. Although data collectors were careful to explain that there will be no material or other gain by participation in the assessment, respondents might have exaggerated or underestimated responses if they believed it would be in their interest to do so. In some instances, (e.g., when interviewing intended beneficiaries), responses might have been constrained due to fear of reporting or stigma such as with questions around SGBV, however, based on our qualitative study, it was noted that SGBV was normalized and responses were less likely to be constrained. Although it is possible that differences within the interviewer due to ethnicity, sex, or overall comfort level during the interview could bias the results, this was mitigated largely through the use of local data collectors, properly trained in interviewing techniques who did not interview in areas they are familiar with. According to most versions of customary law, death does not terminate a marriage. A widow is therefore still considered married to her deceased husband. This made getting true numbers of widow headed households difficult as the data collectors marked them as “married.” Data cannot be extrapolated to represent other counties or the entire State. However, the population of Gogrial West is homogeneous and therefore it is likely the study has practical application to Dinka populations beyond the sampling frame.

## Abbreviations

ANC: Antenatal Care
CRC: Canadian Red Cross
DPT3: Diphtheria, Pertussis, Tetanus × 3 doses (full immunization) ICRC, International Committee of the Red Cross
IPTp: Intermittent Treatment for Malaria in Pregnancy ITN, Insecticide Treat Nets
MNCH: Maternal, Newborn, Child Health
MOGCSW: Ministry of Gender and Child Social Welfare (South Sudan)
SGBV: Sexual and Gender Based Violence
TBAs: Traditional Birth Attendants
TT2+: 2 Doses of Tetanus Toxoid
WASH: Water, Sanitation and Hygiene
WHO: World Health Organization

## References

[1] The Republic of South Sudan: The Sudan Household Health Survey. 2010. http://www.southsudanembassydc.org/PDFs/others/SHHS%20II%20Report%20Final.pdf. Accessed 9 Dec 2015.

[2] United Nations Office of for the Coordination of Humanitarian Assistance. South Sudan Statistics. 2014. https://docs.unocha.org/sites/dms/SouthSudan/South_Sudan_Media_Briefing_Pack/South%20Sudan%20humanitarian%20and%20development%20statistics%20-%20December%202013.pdf. Accessed 4 Aug 2016.

[3] South Sudan National Bureau of Statistics. Key Indicators for Warrap, South Sudan. 2011. http://static1.1.sqspcdn.com/static/f/750842/14504200/1317907538463/Key+Indicators_81.pdf?token=GzB%2BIE L1bLY3IFb%2BGUmfLZpNeWw%3D. Accessed 9 Dec 2015.

[4] Taylor S (2012). Beyond the Health Governance Gap: Maternal, newborn, and child health in South Sudan.

[5] United States Agency for International Development. The Southern Sudan Maternal and Child Health Transformation (MaCHT) Project Operational Research Baseline Report. July. 2013. http://pdf.usaid.gov/pdf_docs/pa00jgmb.pdf. Accessed 9 Dec 2015.

[6] World Medical Association. Declaration of Helsinki Ethical Principles for Medical Research Involving Human Subjects. 2000. http://www.wma.net/en/30publications/10policies/b3/17c.pdf. Accessed 9 Dec 2015. 11122593

[7] Statistical Yearbook for South Sudan. 2010. http://static1.1.sqspcdn.com/static/f/750842/10009744/1293740437687/Statistical+Yearbook+Final.pdf?token=5MnL6vW4kfmLIucQYoHPa1c7%2F1Y%3D. Accessed 9 Dec 2015.

[8] World Health Organization. Facilitator Guide for The EPI Coverage Survey: Training For Midlevel Managers. WHO Expanded Program on Immunization (EPI). 1991;WHO/EPI/MLM/91.11.

[9] Tinker A, Hoope-Bender P, Azfar S, Bustreo F, Bell R (2005). A continuum of care to save newborn lives. Lancet.

[10] Physicians for Human Rights. The Prevalence of Sexual Violence and Other Human Rights Abuses Among Internally Displaced Persons in Sierra Leone: A Population-based Assessment. 2002. https://s3.amazonaws.com/PHR_Reports/sierra-leone-sexual-violence-2002.pdf. Accessed 4 Aug 2016.

[11] Gakidou E, Cowling K, Lozano R, Murray CJL (2010). Increased educational attainment and its effect on child mortality in 175 countries between 1970 and 2009: a systematic analysis. Lancet.

[12] Ahmed S, Qingfeng L, Lui L, Tsui AO (2012). Maternal deaths averted by contraceptive use: an analysis of 172 countries. Lancet.

[13] Darmstadt GL, Bhutta ZA, Cousens S, Adam T, Walker N, De Bernis L (2005). Evidence-based, cost-effective interventions: how many newborn babies can we save?. Lancet.

[14] World Health Organization. Opportunities for Africa’s Newborns. http://www.who.int/pmnch/media/publications/oanfullreport.pdf. Accessed 11 Dec 2015.

[15] Mugo NS, Dibley MJ, Agho KE (2015). Prevalence and risk factors for non-use of antenatal care visits: analysis of the 2010 South Sudan household survey. BMC Pregnancy Childbirth.

[16] Edmond (2006). Delayed Breastfeeding Initiation Increases Risk of Neonatal Mortality. Pediatrics.

[17] World Health Organization. Global and regional estimates of violence against women: Prevalence and health effects of intimate partner violence and non-partner sexual violence. 2013. http://www.who.int/reproductivehealth/publications/violence/9789241564625/en/. Accessed 11 Dec 2015.

[18] Campbell J, Garcia-Moreno C, Sharps P (2004). Abuse During Pregnancy in Industrialized and Developing Countries. Violence Against Women.

[19] Asling-Monemi K, Peña R, Ellsberg MC, Persson LA (2003). Violence Against Women Increases the Risk of Infant and Child Mortality: A Case Study in Nicaragua. Bull World Health Organ.

[20] Government of Southern Sudan. South Sudan’s Constitution of 2011. http://www.sudantribune.com/IMG/pdf/The_Draft_Transitional_Constitution_of_the_ROSS2-2.pdf. Accessed 9 Jan 2017.

[21] Government of Southern Sudan. Child Act 2008. http://www.refworld.org/docid/49ed840c2.html. Accessed 11 Dec 2015.

[22] Adhikari RK, Bott S, Jejeebhoy S, Shah, Puri C (2003). Early marriage and childbearing: risks and consequences. Towards Adulthood: Exploring the Sexual and Reproductive Health of Adolescents in South Asia.

[23] Khan KS, Wojdyla D, Say L, Gulmezoglu AM, Van Look PF (2006). WHO analysis of causes of maternal death: a systematic review. Lancet.

[24] Government of Southern Sudan. Health Sector Development Plan 2011–2015. http://www.gunneweg-imprint-consultants.nl/wp-content/uploads/2011/10/HSSDPL2010-2015-SOUTH-SUDAN.pdf. Accessed 11 Dec 2015.

[25] Government of Southern Sudan. Southern Sudan Maternal, Neonatal, and Reproductive Health Strategy Action Plan (2008–2011). http://www.canwach.ca/wp-content/uploads/2013/09/Southern-Sudan-Maternal-Neonatal-and-Reproductive-Health-Strategy.pdf. Accessed 9 Jan 2017.

[26] Geere J-AL, Hunter PR, Jagals P (2010). Domestic water carrying and its implications for health: a review and mixed methods pilot study in Limpopo Province, South Africa. Environ Health.

[27] Cheng JJ, Shuster-Wallace CJ, Watt S, Newbold BK, Mente A (2012). An ecological quantification of the relationships between water, sanitation and infant, child, and maternal mortality. Environ Health.

[28] Clark S (2004). Early Marriage and HIV Risk in Sub-Saharan Africa. Stud Fam Plann.

[29] World Health Organization. Changing cultural and social norms that support violence. http://www.who.int/violence_injury_prevention/violence/norms.pdf. Accessed 11 Dec 2015.

